# Vitamin B12 Deficiency Observed in Children With First Afebrile Seizures

**DOI:** 10.7759/cureus.13745

**Published:** 2021-03-06

**Authors:** Serkan Kirik, Zekiye Çatak

**Affiliations:** 1 Pediatric Neurology, Fırat University School of Medicine, Elazig, TUR; 2 Department of Medical Biochemistry and Clinical Biochemistry, Elazig Fethi Sekin City Hospital, Elazig, TUR

**Keywords:** vitamin b12, homocysteine, seizure, infantile

## Abstract

Objective: Vitamin B12 deficiency can lead to many different types of neurological symptoms and seizure can be seen as the first symptom. In the present study, we aimed to evaluate patients with seizures who were found to have vitamin B12 deficiency and whose seizures resolved with vitamin B12 treatment.

Methods: A total of 26 infants were included in this retrospective study. The patients were evaluated in terms of clinical findings, laboratory tests including homocysteine, electrophysiological studies, neuroimaging studies, and other neurological examination findings.

Results: Of 26 patients, 14 (53.8%) were male. The mean age of the patients was 8±4.8 months. Sixteen patients had generalized tonic-clonic seizures, and two patients had epileptic spasm (West syndrome)-type seizures. Six patients had abnormal discharge on electroencephalography. Twelve patients had abnormal findings in brain magnetic resonance imaging studies. Homocysteine ​​level was high in all patients at admission.

Conclusion: The presence of seizures, including infantile spasm, is a very important and treatable manifestation of vitamin B12 deficiency. Considering the irreversible sequelae of increased homocysteine, vitamin B12 supplementation administered for an appropriate period and at an appropriate dose both prevents the use of unnecessary antiepileptic drugs and eliminates the need for unnecessary tests and examinations.

## Introduction

Vitamin B12 (VB12) is one of the essential vitamins that affect various systems. Its deficiency causes significant effects on various systems such as the central nervous and hematopoietic system, where cells with rapid mitotic activity are found [1,2]. While its long-term deficiency causes insufficient myelination in the spinal cord and brain, it also causes cerebral atrophy in some patients. In infants, VB12 deficiency may be characterized by signs of developmental delay, such as irritability, apathy, ataxia, anorexia, and mental retardation. More rarely, it can cause seizures, including infantile spasm (IS) [3-5]. Seizures even may appear as the first sign of VB12 deficiency [1,4].

VB12, which is involved in DNA synthesis, is an important cofactor involved in methylation of homocysteine ​​to methionine and conversion of methylmalonyl coenzyme A to succinyl coenzyme A. Factors such as delayed myelination or demyelination of nerves, changes in the S-adenosylmethionine:S-adenosylhomocysteine ratio, neurotrophic imbalance, increased homocysteine ​​and methylmalonic acid, neurotoxic cytokines and accumulation of lactate in brain cells have been implicated in the mechanism of neurological findings and seizures [4-6]. It is very important to see a dramatic improvement in the clinical picture with the regulation of nutritional intake and treatment of VB12 deficiency. In some cases, even if VB12 levels are within the normal range, high homocysteine ​​levels are alerting for VB12 deficiency. Seizures in infancy have a wide variety of clinical presentations, the outcomes of which differ by etiology. Seizures may occur in the form of generalized tonic-clonic (GTC), atonic (AS), myoclonic (MS), focal seizure (FS) or IS [4,5,7,8].

In this study, we report 26 patients presenting with afebrile seizures before 24 months of life, who were found to have VB12 deficiency and whose seizures decreased after VB12 treatment.

## Materials and methods

In this retrospective study, among 702 patients who presented with first afebrile seizure to the Aydin Maternity and Children Hospital between July 2018 and June 2020, 26 pediatric patients aged three to 20 months were retrospectively enrolled after their medical records were reviewed. Demographic, clinical/neurological, and laboratory findings of the patients were obtained from hospital records and screened for abnormalities. Complete blood count, peripheral smear examinations, serum vitamin B12, folic acid, iron, iron binding, ferritin, homocysteine ​​and other biochemical studies were collected in all patients. Since hemoglobin and leukocyte counts vary with age, the diagnosis of anemia was defined as a hemoglobin level <11 g/dL; leukopenia was defined as a white blood cell count <6000 mm3; and thrombocytopenia was defined as a platelet count <150,000 /cmm [6]. Vitamin B12, folic acid, hemogram and other biochemical values ​​of the mothers of 18 patients who were breastfed were analyzed.

Patients with low serum VB12 levels and afebrile seizures due to low VB12 levels were included in the study. Patients with underlying additional metabolic diseases, genetic diseases such as a chromosomal anomaly, structural anomaly of the brain such as hydrocephalus or schizencephaly, folic acid deficiency in addition to VB12 deficiency, intrinsic factor deficiency, history of ileal resection due to intestinal operation, and missing medical records were excluded from the study. Values ​​<200 pg/mL were used to define VB12 deficiency in children [4,7,8]. VB12 level was checked one month and three months after the initiation of VB12 treatment. Homocysteine ​​values ​​were also checked one and three months after B12 treatment. Homocysteine ​​levels >15 µmol/L (5-15 µmol/L) were taken as the threshold for elevated homocysteine.

Electroencephalography (EEG) and brain magnetic resonance imaging (MRI) data were collected from all patients. All patients had control EEG records. EEG was repeated in all patients after the first and sixth months of follow-up in our clinic routinely. Patients’ medical records were reviewed for seizures and additional neurological findings for nine to 12 months. Seizure semiology was determined according to the International League Against Epilepsy (ILAE) 2017 seizure classification, with reference to the video recordings of the patients or the anamnesis provided by the families [9]. Denver-2 developmental screening test was applied to 12 patients with signs of hypotonia in addition to seizures. The patients were treated with intramuscular VB12 at a dose of 50-100 μg every other day for one week, twice weekly for two weeks, and once weekly for another four weeks. In our clinical practice this VB12 treatment schema was used routinely with those admitted to our pediatric neurology department with afebrile seizure or hypotonia. VB12 was also administered to mothers with low B12 levels who were feeding their babies with breast milk. The patient who used antiepileptics for the longest period used it for six months.

Local ethics committee approval was obtained for the study, which was performed in accordance with the Declaration of Helsinki. Written informed consent was obtained from the patients’ families.

Statistical analysis

Statistical analyses were performed using the Statistical Package for the Social Sciences (SPSS) version 20 software package (IBM Corp., Armonk, NY, USA). All quantitative data were expressed as mean±standard deviation. All categorical variables were expressed as number and percentage (n, %).

## Results

Of the 26 patients, 14 (53.8%) were male and 12 (46.1%) were female. The mean age of the patients was 8±4.8 months (three to 20 months). One patient was fed exclusively breast milk, 16 patients were fed breast milk and formula-supplement food. Four (30.7%) patients had stopped breast milk intake at least three months ago. Four (15.3%) patients had a height and weight <3rd percentile. These four patients were exclusively breastfed. Eight (30.7%) patients were 0 to six months old, 14 (53.8%) patients were six to 12 months old, and four (15.3%) patients were 12-24 months old. None of these patients had a head circumference <3rd percentile. The mean serum VB12 level was 152.6±24.3pg/mL (77pg/mL-193pg/mL). Hemoglobin level was low in five (38.4%) patients. Mean corpuscular volume (MCV) was 81.2±7.3fL. Only two patients had pancytopenia. Megaloblastic changes detected in three patients. Hyperhomocysteinemia (>15 mmol/L) was detected in all patients at admission. Homocysteine ​​levels checked in the third month following the VB12 treatment were completely normalized.

An analysis of the data of 18 breastfeeding mothers revealed that VB12 level was low in all of them (163.7pg/mL±16.7 (139pg/ml-197pg/ml)). None of the mothers were vegan, but the families of 14 patients had a low socio-economic level and did not regularly eat foods rich in VB12 such as red meat. Laboratory features are shown in Table 1.

**Table 1:** Laboratory findings of patients with vitamine B12 deficiency and seizures. WBC: white blood cell; MCV: mean corpuscular volume.

Parameters	Mean±SD	Range
Homocysteine (mmol/L)	19.3±4.5	12.7-24.9
Homocysteine (mmol/L) control 1st month (infants)	13.6±4.9	9.4-17.5
Homocysteine (mmol/L) control 3rd month (infants)	9.1±3.7	5.2-13
Vit. B12 (infants) (pg/mL)	152.6±24.3	77-193
Vit. B12 control 1st month (infants) (pg/mL)	712.8±91.3	628.1-814.1
Vit. B12 control 3rd month (infants) (pg/mL)	652.2±62.7	526-743
Vit. B12 (mothers) (pg/mL)	163.7±16.7	139-197
WBC (/mm³)	9.3±3.4	3.0-12.5
Hemoglobin (g/dL)	10.8±1.8	8.9-12.8
Platelet (×10³/mm³)	284.6±154.8	112-551
MCV (fL)	81.2±7.3	74-96

Regarding the seizure semiology of the patients, 16 patients had generalized tonic-clonic (GTC) seizures, four patients had atonic seizures (AS), two patients had myoclonic seizures (MS), two patients had focal seizures (FS) and two patients had IS. Synthetic adrenocorticotropic hormone (ACTH) treatment was administered in addition to VB12 in the patients with IS seizures. The patient who had MS had been started on levetiracetam one week prior. However, his seizures persisted. His VB12 level was found to be low (154pg/mL). VB12 supplementation was initiated for all patients. No seizures were observed in any of the patients in the third month of treatment. All patients' EEG findings were revealed from medical records and EEG abnormalities were detected in six patients. The patients diagnosed with IS had a hypsarrhythmia pattern on the EEG and were diagnosed with symptomatic West syndrome because of the cognitive delay. In addition, two patients with MS and two patients with GTC seizure had epileptic discharges on EEG. All the patients' EEG records checked in the sixth month were within normal limits.

Brain MRI examinations revealed delayed myelination in six patients and cortical atrophy in six patients. A patient with cortical atrophy also had thinning of the corpus callosum. Growth retardation was found in all 12 patients who had the Denver-2 developmental screening test. Significant improvement was observed in the Denver-2 developmental tests performed nine to 12 months later. Table 2 shows the EEG and MRI results as well as the agents used in the treatment.

**Table 2:** Electrophysiological findings, imaging findings and treatment strategies of patients who admitted for seizures secondary to vitamin B12 deficiency. M = Male, F = Female, EEG: Electroencephalography, MRI: Magnetic resonance imaging, GTC: Generalized tonic clonic, AS: Atonic seizure, FS: Focal seizure, MS: Myoclonic seizure, IS: Infantil spasm, N: Normal, DM: Delayed myelination, CA: Cortical atrophy, TCC: Thinnig corpus callosum, LEV: Levetiracetam, ACTH: Adrenocorticotropic hormone, PB: Phenobarbital

Patient no&gender-age	Seizure type	1st. EEG	2nd. EEG	Brain MRI findings	Treatment	Hypotonia
1-F- 10 months	GTC	N	N	N	B12	+
2-F- 16 months	GTC	N	N	N	B12	+
3-M- 6 months	GTC	N	N	DM+CA	B12	-
4-F- 9 months	GTC	Generalized sharp waves	N	CA	B12+PB	+
5-M- 9 months	GTC	N	N	DM	B12	-
6-M- 7 months	GTC	N	N	N	B12	-
7-M- 15 months	GTC	N	N	CA+TCC	B12	-
8-F- 11 months	GTC	N	N	N	B12	-
9-F- 7 months	GTC	N	N	N	B12	+
10-F- 11 months	GTC	N	N	N	B12	+
11-M- 12 months	GTC	N	N	DM+CA	B12	-
12-M- 14 months	GTC	Generalized sharp waves	N	N	B12+PB	+
13-F- 7 months	GTC	N	N	DM	B12	-
14-M- 9 months	GTC	N	N	N	B12	-
15-M - 20 months	GTC	N	N	CA+TCC	B12	-
16-F- 7 months	GTC	N	N	N	B12	-
17-M- 8 months	AS	N	N	CA	B12	-
18-F- 6 months	AS	N	N	N	B12	-
19-M- 9 months	AS	N	N	N	B12	-
20-F - 3 months	AS	N	N	N	B12	-
21-M- 8 months	FS	N	N	N	B12	+
22-M- 12 months	FS	N	N	N	B12	+
23-F - 8 months	IS	Hypsarrhythmia pattern	Sharp waves (bilateral frontal electrodes)	DM	B12+ACTH	+
24-M - 6 months	IS	Hypsarrhythmia pattern	Sharp waves (frontal and occipital electrodes)	DM	B12+ACTH	+
25-F- 17 months	MS	Spike waves in frontal electrodes	N	CA	B12+LEV	+
26-M- 5 months	MS	Spike waves in frontal and central electrodes	N	CA	B12+LEV	+

## Discussion

Afebrile seizures in the infantile period are usually complicated by difficulties in diagnosis and treatment. Although they are less common, benign and treatable forms are of particular importance because in such cases, rapid diagnosis is essential to avoid unnecessary and expensive examinations, prolonged antiepileptic use. VB12 plays a role in DNA synthesis, the deficiency of which causes various symptoms affecting all age groups. Epilepsy is a rare condition in children and adults with VB12 deficiency. The main cause of epileptogenesis due to VB12 deficiency is unclear. This phenomenon may be explained by an increased sensitivity of brain neurons covered with damaged myelin to the stimulating effects of glutamate. Seizures can be of different semiology from GTC seizure to IS [1,3,4]. Roschitz et al. described GTC seizures associated with VB12 deficiency. GTC seizures were the most common seizure type in our patients. Seizure control was achieved with VB12 treatment in all patients who had seizures, and long-term antiepileptic use was not required [10].

Increased homocysteine ​​leads to axonal damage and neurotoxicity by activating N-methyl-D-aspartate receptors. There are no alternative pathways to metabolize homocysteine. Therefore, as VB12 deficiency continues, homocysteine ​​increases, leading to accumulation toxicity, cerebral atrophy, mental retardation and seizures. Homocysteine ​​also acts as an excitatory neurotransmitter that competes with gamma-aminobutyric acid, causing seizures. In such cases, it should be aimed to timely detect VB12 deficiency and to reduce homocysteine ​​level by administering VB12 for an appropriate period [11,12]. Seizures were detected in 12 patients in the study by Taşkesen et al. who reported that seizure control was achieved in patients with hyperhomocysteinemia and VB12 treatment [13]. In our study, the homocysteine ​​level was found to be high in all patients at the time of diagnosis. The homocysteine ​​level measured after VB12 treatment for seven weeks was found to be regressed in all patients. However, although there was a decrease in the homocysteine ​​levels at the end of the first month, hyperhomocysteinemia regressed in all patients after the completion of a seven-week VB12 treatment. Achievement of seizure control and normalization of EEG findings in the third month following the initiation of B12 treatment is very important because it shows that abnormal neuronal discharges are suppressed and seizure control is achieved with the elimination of homocysteine ​​elevation.

Serin et al. reported three patients, one with focal convulsion, one with GTC, and one with IS [4]. The authors reported that the patients were successfully treated with VB12 and ACTH without the need for additional antiepileptic drugs, and all patients remained seizure-free during follow-up. Recent studies show pro-inflammatory cytokines and anti-inflammatory cytokine IL-10 are all involved in the pathogenesis of epilepsy by exacerbating tissue injury, especially in the brain. After initiation of VB12 treatment cytokine activity decreases, preventing further seizures [2,5,6]. In our study, 16 patients had GTC seizures and four patients had AS. In two of these patients, phenobarbital was started because the seizures recurred during the day. These patients had marked hypotonia and cerebral atrophy on brain MRI. Urinary methylmalonic acid excretion was increased in metabolic screening tests. Seizures were subsequently controlled with VB12 treatment in these patients. Phenobarbital treatment was discontinued in six months.

**Figure 1:**
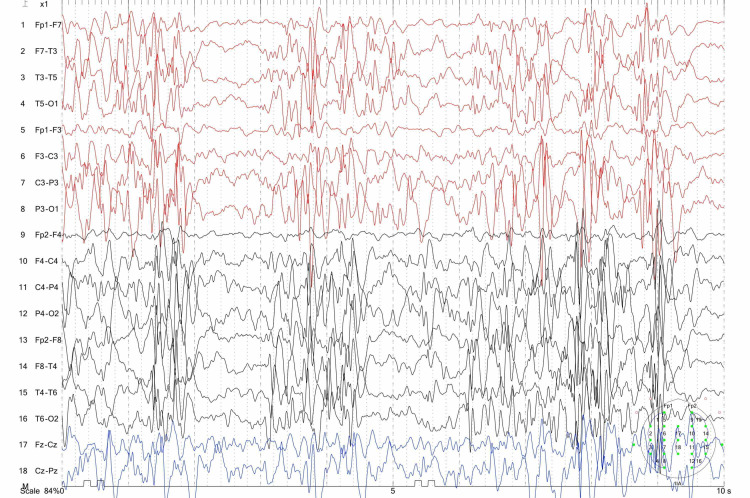
Electroencephalography record of patient with infantile spasm before treatment.

A small number of cases with West syndrome secondary to VB12 deficiency have been reported in the literature [3,14]. Serin et al. presented a six-month-old boy with IS, drowsiness and psychomotor regression diagnosed with West syndrome who improved significantly after VB12 treatment, and his EEG was found to be normal after three months [3]. Our non-contiguous patients were an eight-month-old girl and a six-month-old boy. Both of the patients had a normal neuromotor development initially; they presented to a physician after their families noticed sudden contractions especially when waking up from sleep, as well as a pause and regression in motor skills in the preceding month. Their EEGs revealed a hypsarrhythmia and burst-suppression pattern (Figure 1). After VB12 and ACTH treatment, the spasms disappeared. EEGs were completely normal after six months of treatment. The patients' seizures did not recur during follow-up.

Hypotonia and neurodevelopmental delay are the most common findings in infants who are exclusively breastfed and receive inadequate amounts of VB12 [1-5]. VB12 deficiency is important in the etiology of hypotonic infants as it is a treatable cause. Since evidence of macrocytic anemia can occur without peripheral smear and laboratory findings, it is recommended that patients be examined for this aspect. In Taşkesen et al., hypotonia was the most common neurological finding [13]. Also in our study, hypotonia was the most common neurological finding accompanying seizures. The Denver-2 developmental tests of these patients revealed a delay in the neuromotor steps according to age. After the administration of VB12, hypotonia regressed in the repeated examinations.

The most important cause of VB12 deficiency in infants is insufficient nutritional intake. Malnourished mothers feeding their babies with breast milk have been held responsible for this occurrence. Low socio-economic level of the mother, providing the mother with inadequate health counseling, and misinformation about infant nutrition such as delayed introduction of supplementary foods may be major causes of VB12 deficiency in infants [8,13]. In our study, VB12 level was low in all mothers who fed their infants with breast milk (18 mothers). In addition to infants, VB12 support was provided to mothers. Mothers were informed about the possibility of having a baby with the same disorder in the future and the importance of foods containing VB12 (meat, poultry, eggs, etc.). Positive progress of the examination findings and the absence of seizure recurrence after nine to 12 months despite the seven-week VB12 treatment show the value of the VB12 treatment and nutritional recommendations provided to the mother.

**Figure 2:**
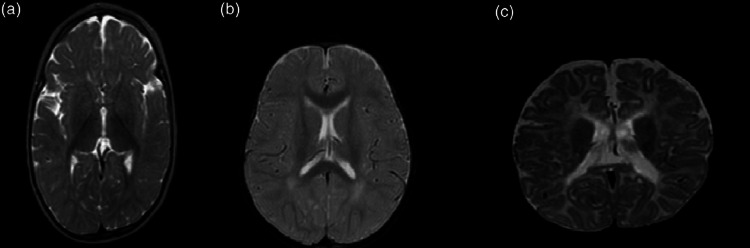
The magnetic resonance imaging findings of the patients. (a) Axial T2-weighted image has mild atrophy in the temporal area and delayed myelination. (b) Axial T2-weighted image has the delayed myelination in the periventricular areas. (c) Axial T2-weighted image shows mild cerebral atrophy and thinning of the corpus callosum.

Cerebral-cerebellar atrophy, subdural effusion, thinning of the corpus callosum and demyelination/delayed myelination are among the neuroimaging findings of VB12 deficiency. Acıpayam et al. reported that cerebral atrophy, retardation in myelinization and subdural effusion were the most common findings in patients with severe VB12 deficiency [6]. Taşkesen et al. declared thinning of the corpus callosum and cortical atrophy were the most common findings [14]. Another study reported an infant with psychomotor retardation who was found to have demyelination due to nutritional VB12 deficiency [15,16]. In our study, the most common findings were cerebral atrophy (eight patients) and delayed myelination (six patients) (Figure 2). Brain MRI findings of 14 patients were normal.

## Conclusions

This study is one of the most comprehensive and largest study evaluating patients presenting with afebrile seizures, a rare finding of nutritional VB12 deficiency, performed in the western region of our country. The treatment should be given for an appropriate period, patients should be followed up frequently, and homocysteine level ​​should be repeated in patients with persistent complaints. VB12 deficiency is particularly a serious problem in countries with low socio-economic level and should be kept in mind for patients presenting with seizures. In this way, unnecessary tests and antiepileptic use can be prevented. With respect to this treatable and preventable condition, it is of great importance to treat breastfeeding mothers and to provide them with appropriate dietary counseling. These important data may be utilized for future proactive strategies in the prevention and treatment of seizures in this population.
